# pSETT4, an Improved φC31-Based Integrative Vector System for *Actinoplanes* sp. SE50/110

**DOI:** 10.1128/MRA.00596-20

**Published:** 2020-09-24

**Authors:** Lena Schaffert, Lucas Jacob, Susanne Schneiker-Bekel, Marcus Persicke, Camilla März, Christian Rückert, Alfred Pühler, Jörn Kalinowski

**Affiliations:** a Microbial Genomics and Biotechnology, Center for Biotechnology, Bielefeld University, Bielefeld, Germany; b Senior Research Group in Genome Research of Industrial Microorganisms, Center for Biotechnology, Bielefeld University, Bielefeld, Germany; University of Maryland School of Medicine

## Abstract

The pSETT4 vector integrates into the *Actinoplanes* sp. SE50/110 chromosome via the bacteriophage φC31 integrase and allows cloning of a gene of interest by Golden Gate assembly (BsaI). T4 terminators surround the expression cassette to isolate the transcriptional unit and to prevent antisense transcription. The system can be used in other *Actinomycetales* by exchanging the promoter.

## ANNOUNCEMENT

*Actinoplanes* sp. SE50/110 (strain ATCC 31044) is known as a natural producer of acarbose (1, 2), which has been used in the treatment of diabetes mellitus since the early 1990s (3, 4). Due to its medical importance, first, genetic tools such as CRISPR/Cas9 (5) and a promoter library (6) were established.

The knowledge gained in previous work (6) was used to develop a novel expression vector, called pSETT4, which will allow easy cloning and overexpression of single genes in *Actinoplanes* sp. SE50/110.

**FIG 1:**
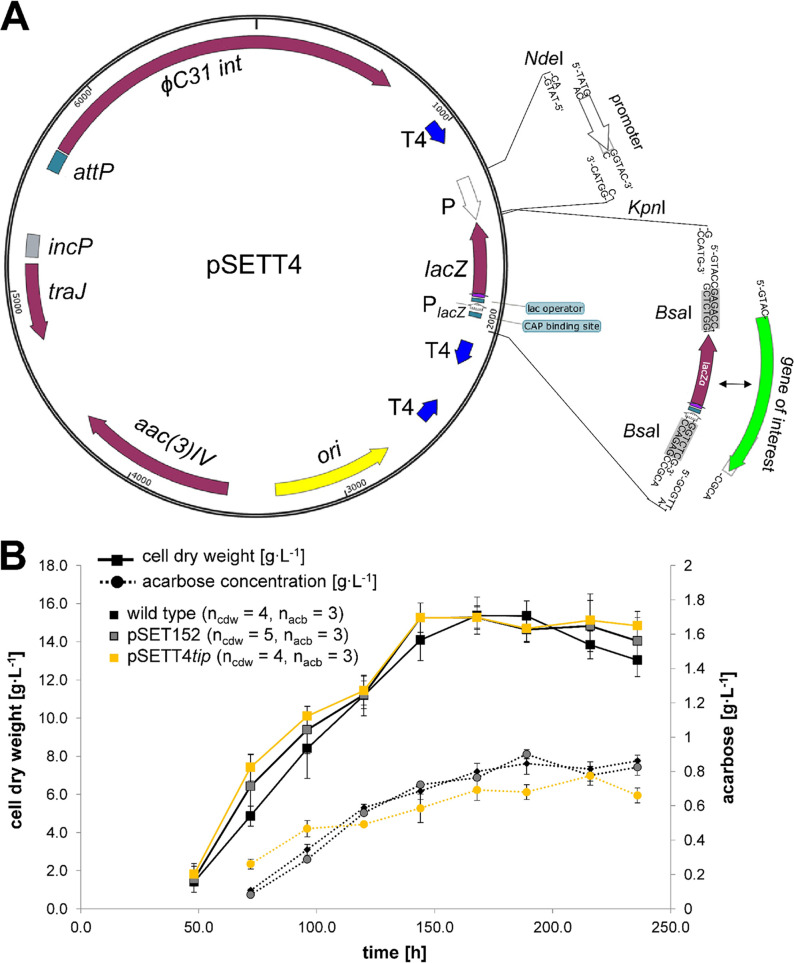
(A) Novel integrative pSETT4 cloning system. The *lacZ′* cassette is flanked by the recognition sites of the restriction enzyme BsaI. BsaI enables exchange of *lacZ* by the gene of interest using Gibson assembly, restriction/ligation cloning, or Golden Gate cloning. As strong expression needs strong termination, T4 terminators were introduced before and after the cloning site. Behind the cloning site, two antiparallel-oriented T4 terminators prevent read-through from both directions. For exchange of the promoter sequence, NdeI and KpnI restriction sites were introduced. Furthermore, the vector contains the integrase gene *int* and the attachment site *attP* of the phage φC31, the origin of transfer (*incP*) and relaxosome gene *traJ*, the high-copy-number ColE1 origin of replication, and the apramycin resistance gene *aac(3)-IV*. (B) Growth and acarbose formation of *Actinoplanes* sp. SE50/110 (pSETT4*tip*), *Actinoplanes* sp. SE50/110 (pSET152), and the wild-type *Actinoplanes* sp. SE50/110 took place in a shake flask in maltose minimal medium. Numbers of replicates are indicated by the *n* values shown in parentheses for both the cell dry weight (cdw) and the acarbose concentration (acb).

For this, the strong promoter of the gene *gapDH* from Eggerthella lenta (6, 7) was cloned in front of a *lacZ′* cassette in a pSET152 backbone (Fig. 1A). The gene *lacZ′* is transcribed under the control of the *lac* promoter and flanked by the recognition sites of the type IIS restriction enzyme BsaI, which enables seamless Golden Gate cloning (8). This way, the cloning effort and time were substantially decreased. In addition, cloning via Gibson assembly (9) and restriction/ligation is also possible.

To isolate the transcriptional units, T4 terminators were introduced before and after the cloning cassette (Fig. 1A). T4 terminators have already been successfully used in the pGUS-cloning system (10). They were proven to block transcription efficiently and prevent read-through from the integrase gene into the gene of interest by whole-track transcriptome sequencing (RNA-seq) analysis (11). By sequencing of native 5′ ends of transcripts derived from a previous promoter-screening experiment (6), two putative antisense promoters were identified behind the gene of interest in antisense orientation in the original vector backbone pSET152 (11), which were removed in the novel system. An additional (third) T4 terminator was introduced behind the cloning side in the opposite orientation to prevent further antisense reads (Fig. 1A). The vector is named pSETT4*gap.*

To allow exchange of the promoter sequence, NdeI and KpnI restriction sites were introduced (Fig. 1A). Here, the medium-strong promoter of *tipA* from Streptomyces lividans (6, 12) was cloned by restriction/ligation cloning, and the vector was named pSETT4*tip.*

For construction of pSETT4*gap*, the cassette, consisting of the *gapDH* promoter, a *lacZ′* gene under the control of the *lac* promoter, and several restriction sites flanked by three T4 terminators, was obtained in three string DNAs (Integrated DNA Technologies, Coralville, IA, USA), assembled by gene splicing by overlap extension (gene SOEing) (13), and cloned into a PCR-linearized backbone using Gibson assembly (9) according to a protocol from reference 6 and using the primers in Table 1.

**TABLE 1:** Sequencing and Gibson assembly primers for the assembly of the novel expression system pSETT4

Fragment/utilization	Template(s)	Size (bp)	Primer sequence (5′–3′)
seq1	pSETT4		TGACCCCATGCCGAACTCAGAAGTGAAACG
seq2			GTACTTCGTCGTGAAGGTCATGACACCATTATAACGAACG
pSET152_lin	pSET152	5,114	CTACGGTGCCGCTTACCGGgctcactcaaaggcggtaatacgg
			CAGACGTCAGCGACGACAGAGaaccatcggcgcagctatttac
genesoeing_for	IDT-orders 1 and 2	1,473	CTCTGTCGTCGCTGACGTCTG
genesoeing_1r			CAGATCTGGAGTCGGTCTAATTT
genesoeing_2f	IDT-orders 2 and 3	878	AGGGTTTTCCCAGTCACGACG
genesoeing_rev			CCGGTAAGCGGCACCGTAG
*tipA*_GAF	pSETGUS	146	GTGGCCCATGCGAGAGTACAATCCCTAGAACGTCCGGG
*tipA*_GAR			TCAACATAAGGTCTCGGTACCATCGGAATACCTCCGTTGCT

For exchange of the promoter, pSETT4*gap* was digested with NdeI and KpnI and treated with shrimp alkaline phosphatase following the supplier’s instructions (Thermo Fisher Scientific, Waltham, USA). The *tipA* promoter was amplified from pSETGUS (10) (Table 1) and assembled with the linearized backbone using Gibson assembly (9).

The cloning mixtures were transferred to Escherichia coli DH5αMCR (14) and selected on Luria/Miller broth medium with 15 g · liter^−1^ agar-agar and 50 mg · liter^−1^ apramycin sulfate. Positive colonies were tested with Sanger sequencing at our in-house sequencing core facility and transferred to *Actinoplanes* sp. SE50/110 by conjugation (6).

The novel expression system pSETT4*tip* displays growth behavior and an acarbose-producing phenotype similar to those of the wild type and the empty vector control carrying pSET152 (Fig. 1B). The cultivation and acarbose quantification were carried out as described before (6).

### Data availability.

The complete sequences of pSETT4*gap* and pSETT4*tip* have been deposited at Addgene under the accession numbers 153413 and 153414. The resources can be obtained from the Addgene depository (https://www.addgene.org/).
